# MiR-146b-5p overexpression attenuates stemness and radioresistance of glioma stem cells by targeting HuR/lincRNA-p21/β-catenin pathway

**DOI:** 10.18632/oncotarget.9214

**Published:** 2016-05-06

**Authors:** Wei Yang, Hongquan Yu, Yueming Shen, Yingying Liu, Zhanshan Yang, Ting Sun

**Affiliations:** ^1^ School of Radiological Medicine and Protection, Medical College of Soochow University, Collaborative Innovation Center of Radiation Medicine of Jiangsu Higher Education Institutions, Soochow University, Suzhou, Jiangsu 215123, China; ^2^ Department of Neurosurgery of The First Affiliated Hospital of Jilin University, Changchun, Jilin 130021, China; ^3^ Neurosurgery and Brain and Nerve Research Laboratory, The First Affiliated Hospital of Soochow University, Suzhou, Jiangsu 215006, China

**Keywords:** microRNA-146b-5p, glioma stem cells, lincRNA-p21, HuR, β-catenin

## Abstract

A stem-like subpopulation existed in GBM cells, called glioma stem cells (GSCs), might contribute to cancer invasion, angiogenesis, immune evasion, and therapeutic resistance, providing a rationale to eliminate GSCs population and their supporting niche for successful GBM treatment. LincRNA-p21, a novel regulator of cell proliferation, apoptosis and DNA damage response, is found to be downregulated in several types of tumor. However, little is known about the role of lincRNA-p21 in stemness and radioresistance of GSCs and its regulating mechanisms. In this study, we found that lincRNA-p21 negatively regulated the expression and activity of β-catenin in GSCs. Downregulation of lincRNA-p21 in GSCs was resulted from upregulation of Hu antigen R (HuR) expression caused by miR-146b-5p downregulation. MiR-146b-5p overexpression increased apoptosis and radiosensitivity, decreased cell viability, neurosphere formation capacity and stem cell marker expression, and induced differentiation in GSCs. Moreover, knock-down lincRNA-p21 or HuR and β-catenin overexpression could rescue the phenotypic changes resulted from miR-146b-5p overexpression in GSCs. These findings suggest that targeting the miR-146b-5p/HuR/lincRNA-p21/β-catenin signaling pathway may be valuable therapeutic strategies against glioma.

## INTRODUCTION

A growing body of evidence suggest that a stem-like subpopulation existed in glioblastoma multiforme (GBM) cells, named glioma stem cells (GSCs), should be responsible for tumor growth, resistance to therapy and recurrence [1–4]. GSCs represent notable similarities to normal neural stem cells, such as the self-renew capacity, stem cell markers (Sox2 and Nestin) expression and differentiation potential [5, 6]. GSCs grow like nonadherent spheres in serum-free medium and showed potent tumor formation ability in immuno-compromised mice [7]. Furthermore, they contribute to cancer invasion, angiogenesis, immune evasion, and therapeutic resistance, providing a rationale to eliminate GSCs population and their supporting niche for successful GBM treatment [8–11]. Elucidation of the molecular mechanisms underlying the therapeutic resistance of GSCs is a critical step to identify novel targets of therapeutic intervention.

Long intergenic non-coding RNAs (lincRNAs) function as transcription and post-transcription regulator governing mRNA splicing, translation and degradation [12, 13]. Emerging evidence suggests that lincRNA-p21, about 3.0 kb in length and located nearly 15 kb upstream of p21/Cdkn1a gene, is a new regulator of cell cycle, apoptosis and DNA repair, and plays a vital role in some diseases including cancer [14–17]. As a p53-induced lincRNA, lincRNA-p21 employs the heterogeneous nuclear ribonucleoprotein K (hnRNP-K) protein to oppress transcription of p53 target gene [18]. Since lincRNA-p21 is downregulated in some kinds of cancer, it might serve as a tumor suppressor [19–21]. Nevertheless, we know little about the pathological function of lincRNA-p21 in glioma cells and GSCs.

It was reported that lincRNA-p21 negatively regulated β-catenin translation at the post-transcriptional level in HeLa cells [22]. Wnt/β-catenin pathway contributes to glioma cells and GSCs proliferation [23, 24]. Chen et al. reported that glioma stem cell-like phenotype was associated with Wnt/β-catenin signal activation [25]. Recently, wang et al. demonstrated that lincRNA-p21 increased colorectal cancer radiosensitivity through deactivating β-catenin pathway [19]. However, whether lincRNA-p21 could inhibit Wnt/β-catenin pathway and function as an radiosensitizer in glioma cells and GSCs remains unknown.

In this study, we investigated the function of lincRNA-p21 in stemness and radioresistance of GSCs and its regulating mechanisms. Our data indicated that lincRNA-p21 was downregulated, while β-catenin was upregulated in GBM and GSCs cell lines and GBM tumor tissues. LincRNA-p21 could negatively regulate β-catenin expression and activity in GBM, particularly in GSCs. Downregulation of lincRNA-p21 in GSCs was resulted from upregulation of Hu antigen R (HuR) expression caused by miR-146b-5p downregulation. MiR-146b-5p overexpression attenuates stemness and radioresistance of GSCs by targeting HuR/lincRNA-p21/β-catenin pathway. Moreover, knock-down lincRNA-p21 or HuR and β-catenin overexpression could rescue the decreased stemness and radioresistance resulted from miR-146b-5p overexpression in GSCs.

## RESULTS

### LincRNA-p21 level is low in GBM

**Figure 1:**
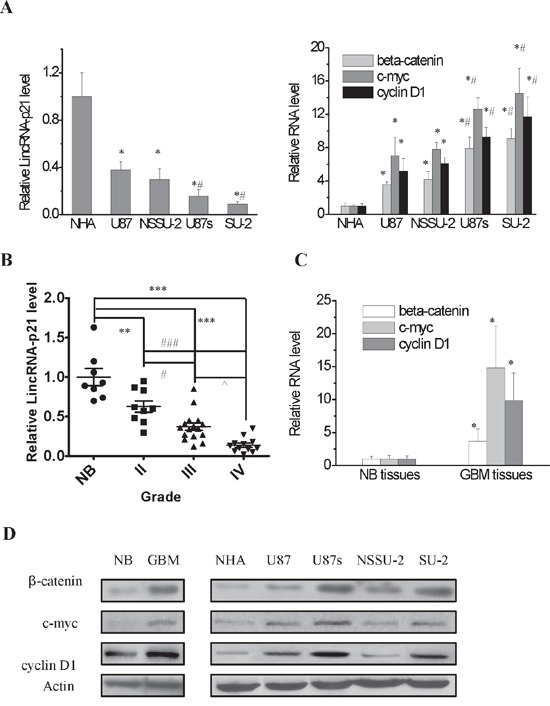
LincRNA-p21 expression level and Wnt/β-catenin signaling pathway activity in GBM and GSCs cell lines and GBM tissues **A.** Real-time RT-PCR analysis of lincRNA-p21, β-catenin and its target genes expression levels in GBM and GSCs cell lines. *P<0.01 vs NHA, ^#^*P*<0.01 vs U87 or NSSU-2. **B.** LincRNA-p21 expression level in glioma tissues and normal brain tissues. **P<0.01, ***P<0.001 vs normal brain tissues; #*P*<0.05, ###*P*<0.001 vs Grade II; ^*P*<0.05 vs Grade III **C.** Real-time RT-PCR analysis of β-catenin and its target genes expression levels in GBM tissues and adjacent normal brain tissues. *P<0.01 vs normal brain tissues. **D.** Detection of protein expression of β-catenin and its target genes in GBM and GSCs cell lines and GBM tissues by Western blot.

First we compared lincRNA-p21 level among normal human astrocytes, GBM and GSCs cell lines to study the possible role of lincRNA-p21 in GBM. The results showed that lincRNA-p21 expression in GBM and GSCs cell lines were lower than normal astrocytes cell line NHA. In addition, lincRNA-p21 expression in GSCs were lower than GBM cell lines (Figure 1A). Then, we compared lincRNA-p21 expression in 37 glioma tissues, including 9 low grade astrocytomas (LGA, Grade II), 16 anaplastic astrocytomas (AA, Grade III) and 12 glioblastoma multiforme (GBM, Grade IV), with 8 normal brain tissues. Our results showed that lincRNA-p21 level in glioma tissues was declined compared to normal brain. In addition, lincRNA-p21 level decreased with the grade of glioma (Figure 1B). Our data indicat that lincRNA-p21 is downregulated in GBM, which might play an important role in GBM initiation and resistance to therapy.

### β-catenin is upregulated in GBM and GSCs cell lines and GBM tumor tissues

It has been reported that lincRNA-p21 inhibited β-catenin translation in cervical cancer cells [22]. Nonetheless, whether lincRNA-p21 could regulate β-catenin expression in GBM still needs to be identified. We first examined β-catenin level in GBM and GSCs cells as well as normal human astrocytes. The results demonstrated that β-catenin expression was higher in GSCs, at mRNA and protein level, than GBM and NHA cells. In addition, β-catenin expression in GBM cell lines was higher than NHA cells (Figure 1A and 1D). Consistently, β-catenin mRNA and protein levels of GBM tissues were higher than normal brain (Figure 1C and 1D). Then, we investigated Wnt/β-catenin signal activity in these cell lines through detecting c-myc and cyclin D1 levels, which are the targets of β-catenin. Our results showed that their mRNA and protein levels were enhanced in GBM and GSCs cells compared to NHA cells (Figure 1A and 1D). Wnt/β-catenin signal activity was also detected in GBM tissues and normal brain. We mixed the total RNA of 12 GBM and 8 normal brain tissues, respectively. The qRT-PCR results demonstrated that c-myc and cyclin D1 mRNA levels in GBM tissues were higher than normal brain (Figure 1C). Consistently, their protein levels were elevated in GBM tissues compared to normal brain (Figure 1D). Our data showed that lincRNA-p21 and β-catenin levels in GBM cells and tissues were inversely correlated, which suggested that lincRNA-p21 might negatively regulate β-catenin signal activity in GBM cells, particularly in GSCs.

### LincRNA-p21 overexpression suppressed Wnt/β-catenin signal activity in GSCs

**Figure 2:**
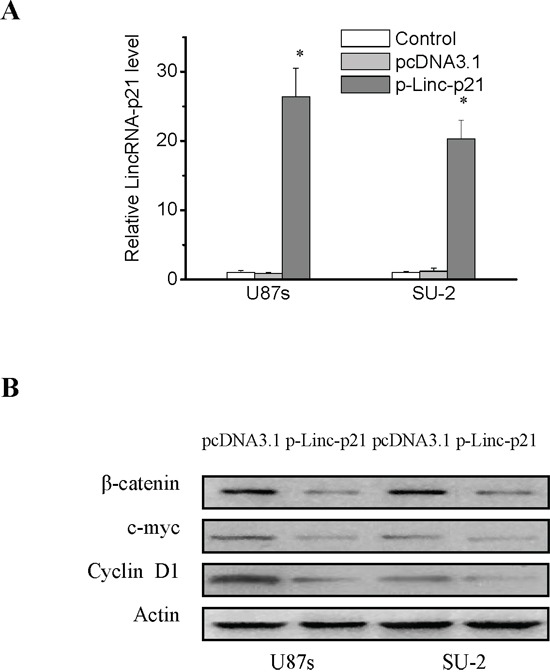
LincRNA-p21 overexpression decreased β-catenin expression and activity in GSCs **A.** Real-time RT-PCR analysis of lincRNA-p21 expression levels in GSCs transfected with lincRNA-p21 overexpression recombinant vectors. *P<0.01 vs Control. **B.** Detection of protein expression of β-catenin and its target genes in GSCs with lincRNA-p21 overexpression by Western blot.

We hypothesized that lincRNA-p21 overexpression might suppress Wnt/β-catenin signaling activity in GSCs, so the lincRNA-p21 overexpression recombinant vectors were constructed and were transfected into GSCs to detect its effect. The results showed that lincRNA-p21 overexpression in GSCs decreased Wnt/β-catenin signaling activity, as proved by downregulation of β-catenin targets (Figure 2A and 2B).

### Regulation mechanisms of lincRNA-p21 expression

**Figure 3:**
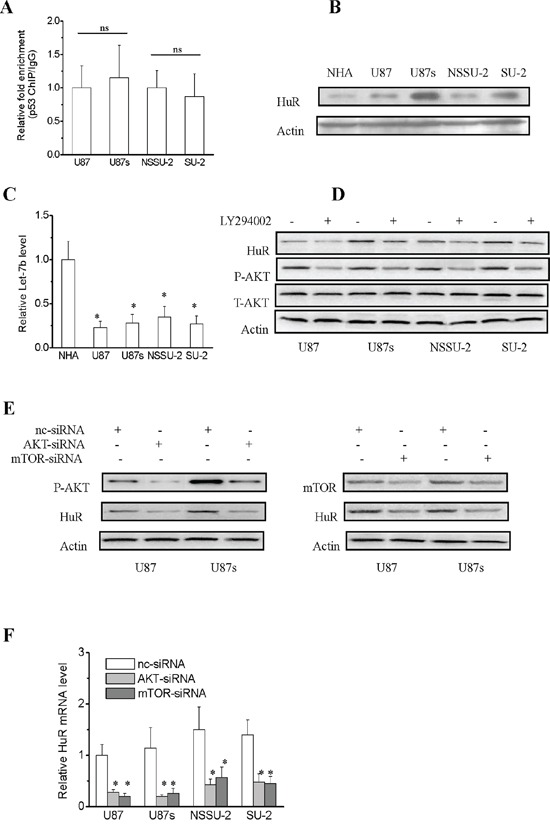
Regulation mechanisms of lincRNA-p21 expression **A.** p53 enrichment at the human lincRNA-p21 promoter in GSCs and non-GSCs glioma cells as quantified by Real-time RT-PCR. The binding efficiency of p53 and lincRNA-p21 promoter in GSCs showed no significant difference compared to the corresponding non-GSCs glioma cells. **B.** Detection of HuR expression levels in GSCs and non-GSCs glioma cells by Western blot. **C.** Real-time RT-PCR analysis of let-7b expression levels in GSCs and non-GSCs glioma cells. *P<0.01 vs NHA. **D.** Transcriptional activation of HuR through PI3K/AKT pathway in both GSCs and non-GSCs glioma cells. **E.** HuR protein expression levels were reduced in both GSCs and non-GSCs glioma cells after AKT and mTOR-siRNA transfection. **F.** Real-time RT-PCR analysis of HuR mRNA levels in both GSCs and non-GSCs glioma cells after AKT and mTOR-siRNA transfection. *P<0.01 vs nc-siRNA.

**Figure 4:**
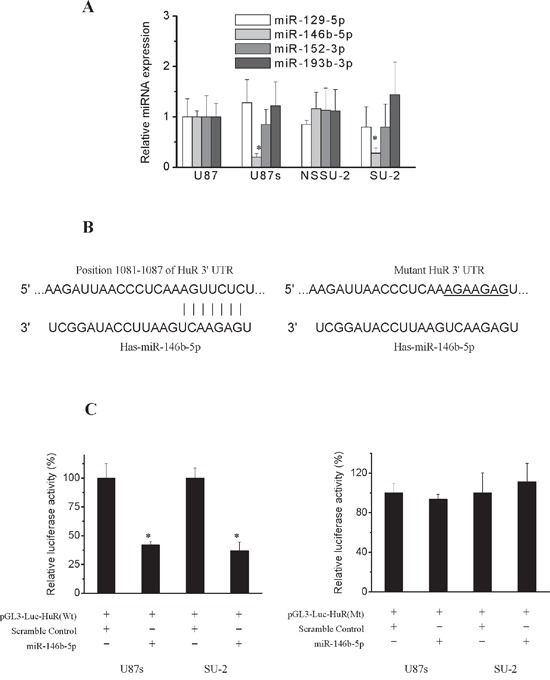
Post-transcriptional regulation of HuR **A.** Real-time RT-PCR analysis of the expression levels of the putative miRs that could target HuR. *P<0.01 vs U87 cells. **B.** A putative miR-146b-5p target site in the wild type and mutated 3′UTR of HuR. **C.** Relative luciferase activity in GSCs transfected with pGL3-Luc vector containing wild type and mutated 3′UTR of HuR. *P<0.01 vs cells transfected with scramble oligos.

To explore the possible mechanisms involved in the lincRNA-p21 downregulation in GSCs, first we examined the transcriptional regulation of lincRNA-p21 in GSCs and non-GSCs glioma cells. LincRNA-p21 is transcriptionally regulated by p53 binding to the p53-binding motif (GGACATGCCCGGGCATGTC) in its promoter [18], so we hypothesized that the interaction between p53 and lincRNA-p21 might be different in GSCs and non-GSCs glioma cells. To test this, we performed chromatin immunoprecipitation analysis to determine whether the binding efficiency of p53 to lincRNA-p21 promoter was different between GSCs and non-GSCs glioma cells. As shown in Figure 3A, the binding efficiency was similar between GSCs and non-GSCs glioma cells, which suggested that downregulation of lincRNA-p21 in GSCs might not result from the transcriptional regulation by p53. In addition to transcriptional regulation, lincRNA-p21 is also subject to post-transcriptional regulation. It is well documented that lincRNA-p21 levels are regulated by the cooperation of mRNA-binding protein HuR, let-7b and Ago2 protein [22]. We next compared HuR and let-7b expression levels in GSCs with non-GSCs glioma cells. As shown in Figure 3B and 3C, non-GSCs glioma cells displayed significantly lower HuR protein levels than GSCs, while let-7b level showed no significant change between GSCs and non-GSCs glioma cells, which suggested that downregulation of lincRNA-p21 in GSCs might result from upregulation of HuR expression. It was reported that HuR is activated through the PI3K/AKT/NF-κB pathway in gastric cancers and through the mTOR/HSF1 pathway in breast cancer cells [26, 27]. Therefore, we next examined whether elevation of HuR levels was associated with transcriptional activation of the gene through the PI3K/AKT pathway. The level of HuR and P-AKT in both GSCs and non-GSCs glioma cells were markedly reduced by the PI3K inhibitor LY294002 (Figure 3D). Furthermore, the mRNA and the protein level of HuR were markedly reduced in both GSCs and non-GSCs glioma cells after AKT and mTOR-siRNA transfection (Figure 3E and 3F). The results demonstrated that HuR is activated through the PI3K/AKT/mTOR pathway in both GSCs and non-GSCs glioma cells, which suggested that elevation of HuR levels in GSCs compared to non-GSCs glioma cells might be not due to transcriptional regulation. In addition to transcriptional regulation, HuR is also subject to post-transcriptional regulation, which occurs chiefly via microRNAs (miRs) and is poorly studied in glioma cells [28–30]. Human HuR 3′ UTR was bio-informaticly analysised for miRNA seed sequences by “starBase v2.0”, which integrates data from five predicted softwares (TargetScan, PicTar, PITA, miRanda/mirSVR and RNA22) [31, 32]. We first choosed miRs whose expression levels showed significant alterations in GBM samples. Then, we selected the common miRs listed in all softwares of starBase. Based on these standard, we screened out 4 miRs: miR-129-5p, 146b-5p, 152-3p and 193b-3p. To determine potential miRNAs which regulate HuR expression in GSCs and non-GSCs glioma cells, we first examined the expression level of the above mentioned miRs by real-time PCR. As shown in Figure 4A, endogenous level of miR-146b-5p was remarkablely declined in GSCs compared with non-GSCs glioma cells, however, level of miR-129-5p, 152-3p and 193b-3p showed no obvious change between GSCs and non-GSCs glioma cells. As miRs are believed to inversely control mRNA translation, miR-146b-5p were selected as a candidate miR targeting HuR and we speculated that the enhanced HuR expression in GSCs might result from the reduced level of miR-146b-5p. In order to identify whether miR-146b-5p targets HuR by binding to its 3′ UTR sequence (Figure 4B), we employed luciferase reporter assays. Generally, a declined luminescence indicated that the 3′-UTR was effectively bound and targeted by the miRNA [33, 34]. As shown in Figure 4C, after miR-146b-5p oligos were transfected into GSCs with the wild type reporter construct pGL3-Luc-HuR, luciferase activity was significantly decreased compared with scramble oligos transfection, whereas mutations in predicted target site of 3′-UTR of HuR gene abrogated the inhibition by miR-146b-5p oligos. These results suggested that HuR might be a target gene of miR-146b-5p in GSCs. Collectively, these data demonstrated that miR-146b-5p was downregulated in GSCs compared to that of non-GSCs glioma cells, which contributed to enhanced HuR expression, resulting in the downregulation of lincRNA-p21 expression, which led to Wnt/β-catenin signaling activation in GSCs.

### MiR-146b-5p overexpression suppressed Wnt/β-catenin signaling activity by targeting HuR/lincRNA-p21 pathway in GSCs

**Figure 5:**
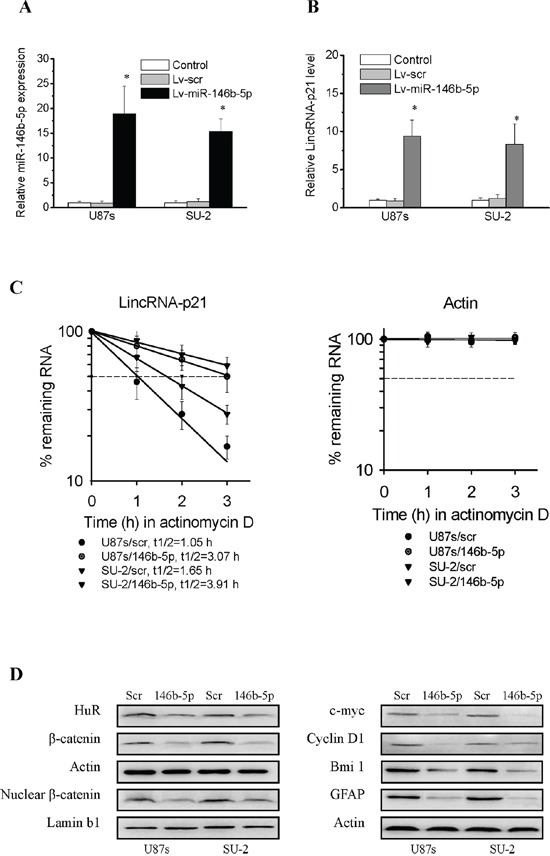
MiR-146b-5p overexpression suppressed Wnt/β-catenin signaling activity through targeting HuR/lincRNA-p21 pathway in GSCs **A.** Detection of miR-146b-5p expression in GSCs with stable integration of miR-146b-5p by real-time PCR. *P<0.01 vs Control. **B.** Detection of lincRNA-p21 expression in GSCs with miR-146b-5p overexpression by real-time PCR. *P<0.01 vs Control. **C.** LincRNA-p21 had slower decay in GSCs with miR-146b-5p overexpression. **D.** Detection of protein expression of β-catenin and its target genes in GSCs with miR-146b-5p overexpression by Western blot.

We hypothesized that miR-146b-5p overexpression might suppressed β-catenin expression and activity in GSCs through targeting HuR/lincRNA-p21 pathway. Lentivirus encoding either miR-146b-5p or scramble sequence were transfected into GSCs to generate stable cell lines. First miR-146b-5p levels in GSCs were detected by real-time PCR. As shown in Figure 5A, miR-146b-5p levels in GSCs with miR-146b-5p integration were higher than control cells, while miR-146b-5p levels in GSCs with scramble sequence integration showed no obvious changes compared with control cells. We next investigated whether miR-146b-5p overexpression could reverse the enhanced HuR expression in GSCs. As shown in Figure 5B, 5C and 5D, miR-146b-5p overexpression markedly reduced HuR expression and decay of HuR target lincRNA-p21 leading to increased lincRNA-p21 expression in GSCs. We further investigated the effect of miR-146b-5p overexpression on β-catenin signaling activity in GSCs. As shown in Figure 5D, miR-146b-5p overexpression significantly decreased cytoplasmic and nuclear β-catenin expression as well as β-catenin targets in GSCs. It was reported that c-myc was an important stem cell regulator [35–37]. Therefore, we further investigated the effect of miR-146b-5p overexpression on GSCs stemness. As shown in Figure 5D, the stem cell marker Bmi1 was significantly decreased, while the differentiation marker glial fibrillary acidic protein (GFAP) was significantly increased in GSCs with miR-146b-5p overexpression, which suggested that miR-146b-5p overexpression decreased GSCs stemness. Our results demonstrated that miR-146b-5p overexpression could suppress Wnt/β-catenin signaling activity through targeting HuR/lincRNA-p21 pathway in GSCs.

### MiR-146b-5p overexpression reduced GSCs viability and arrested GSCs in G0/G1 phase

**Figure 6:**
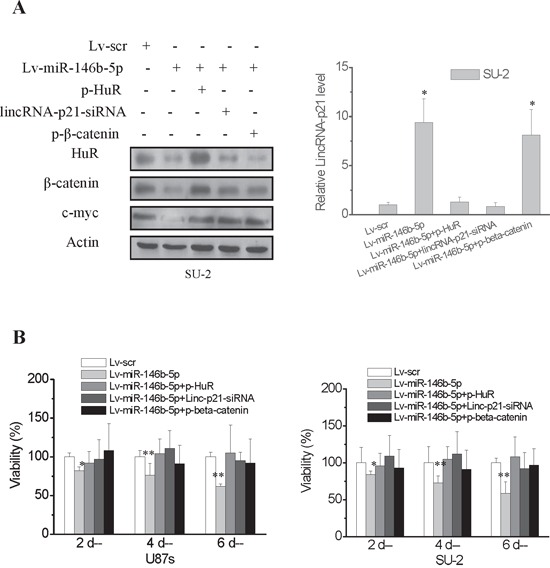
MiR-146b-5p overexpression decreased viability of GSCs The effect of miR-146b-5p overexpression on cell viability of GSCs was investigated *in vitro* by MTT assay. Cell viability was detected on 2, 4 and 6 days after GSCs/Lv-miR-146b-5p transfected with p-HuR, p-β-catenin and lincRNA-p21-siRNA. **A.** The protein levels of HuR, β-catenin and c-myc as well as RNA levels of lincRNA-p21 of GSCs/Lv-miR-146b-5p transfected with p-HuR, p-β-catenin and lincRNA-p21-siRNA were detected 2 days after transfection. *P<0.01 vs Lv-scr. **B.** MiR-146b-5p overexpression decreased GSCs cell viability. *P<0.05, **P<0.01 vs Lv-scr.

**Figure 7:**
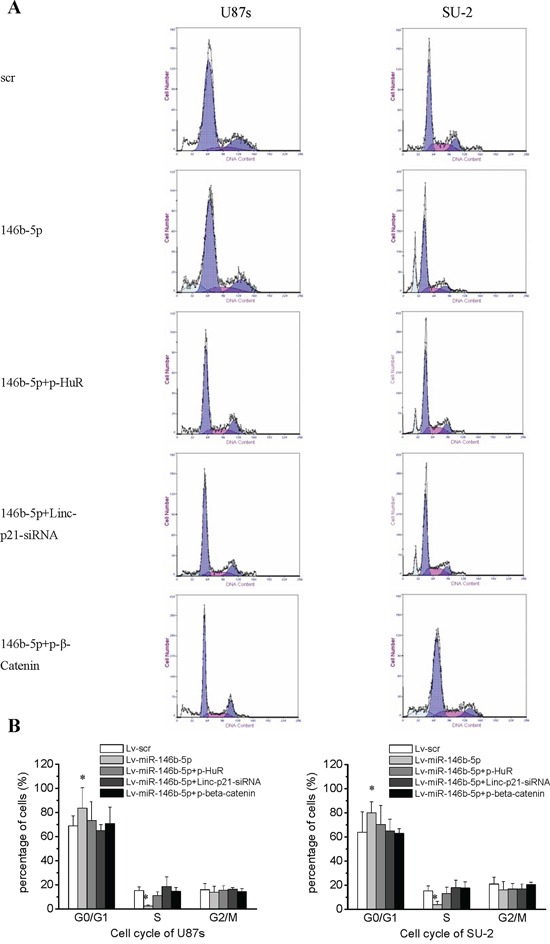
GSCs with miR-146b-5p overexpression arrested in the G0/G1 phase **A.** Cell cycle of GSCs analyzed by flow cytometry. **B.** Cell cycle arrest caused by miR-146b-5p overexpression was rescued by HuR and β-catenin overexpression or lincRNA-p21 knock-down in GSCs.*P<0.01 vs Lv-scr.

The effect of miR-146b-5p overexpression on GSCs viability was detected using MTT assay. GSCs viability was assayed on 2, 4 and 6 days after GSCs/Lv-miR-146b-5p transfected with p-HuR, p-β-catenin and lincRNA-p21-siRNA. The protein levels of HuR, β-catenin and c-myc as well as RNA levels of lincRNA-p21 of these cells were detected 2 days after transfection. C-Myc expression were rescued in GSCs/Lv-miR-146b-5p transfected with p-HuR, p-β-catenin and lincRNA-p21-siRNA. Both HuR overexpression and lincRNA-p21-siRNA decreased the elevated lincRNA-p21 level in GSCs/Lv-miR-146b-5p (Figure 6A). MiR-146b-5p overexpression decreased U87s and SU-2 cell viability and the lowest viability was 61.8% and 58.8% after 6 days culture, respectively (Figure 6B; *P*<0.05 or 0.01). Our data demonstrated that miR-146b-5p overexpression decreased viability of GSCs. However, after p-HuR, p-β-catenin and lincRNA-p21-siRNA transfection, cell viability of GSCs/Lv-miR-146b-5p showed no significant change compared with GSCs with scramble sequence integration, which suggested that knock-down lincRNA-p21 or HuR and β-catenin overexpression could rescue cell viability of GSCs with miR-146b-5p overexpression. To study the mechanism of miR-146b-5p mediated inhibition of cell proliferation, we analyzed GSCs cell cycle using flow cytometry. After 2 days culture, more G0/G1 phase cells and less S phase cells were found in GSCs/Lv-miR-146b-5p compared to GSCs/Lv-scr (Figure 7A and 7B; *P*<0.01). The results showed that miR-146b-5p overexpression arrested GSCs in G0/G1 phase. Nevertheless, the cell cycle arrest induced by miR-146b-5p overexpression also was rescued by HuR and β-catenin overexpression or lincRNA-p21 knock-down in GSCs.

### MiR-146b-5p overexpression increased apoptosis of GSCs

**Figure 8:**
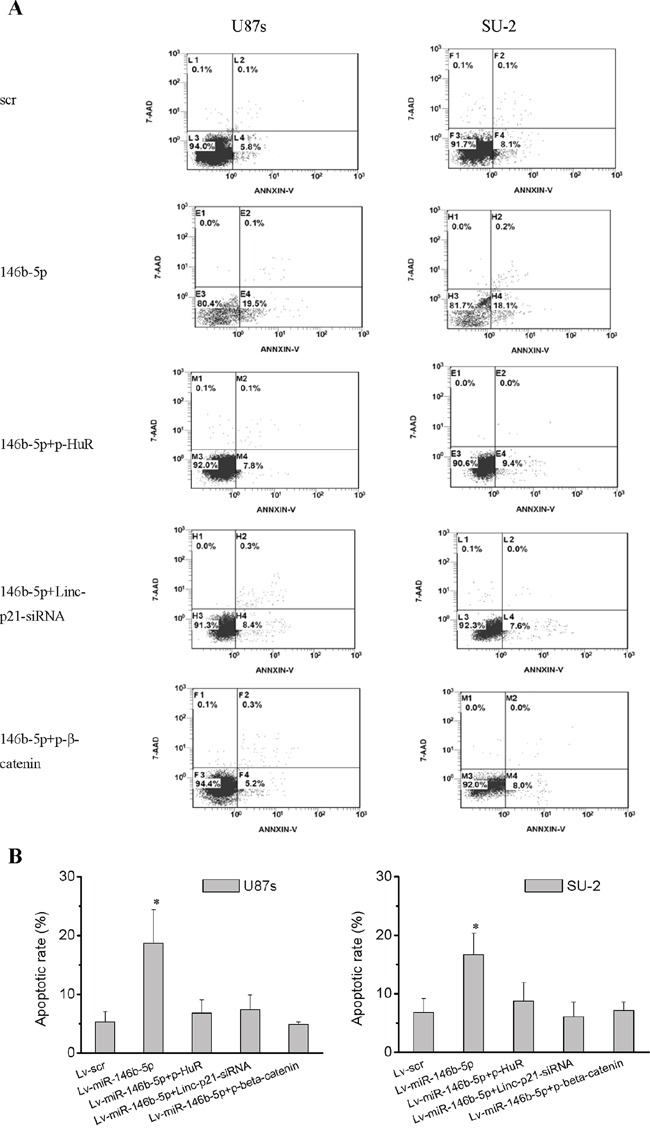
MiR-146b-5p overexpression increased apoptosis of GSCs **A.** Apoptosis of GSCs analyzed by flow cytometry. **B.** Knock-down lincRNA-p21 or HuR and β-catenin overexpression rescued apoptosis of GSCs with miR-146b-5p overexpression. *P<0.01 vs Lv-scr.

GSCs apoptosis was detected by flow cytometry 48 h after p-HuR, p-β-catenin and lincRNA-p21-siRNA transfection. The data indicated that apoptosis of GSCs with miR-146b-5p overexpression was significantly increased compared with GSCs with scramble sequence integration (Figure 8A and 8B). However, after p-HuR, p-β-catenin and lincRNA-p21-siRNA transfection, apoptotic rate of GSCs/Lv-miR-146b-5p showed no significant change compared with that of GSCs/Lv-scr, which suggested that knock-down lincRNA-p21 or HuR and β-catenin overexpression could rescue apoptosis of GSCs with miR-146b-5p overexpression.

### MiR-146b-5p overexpression reduced GSCs neurosphere formation

**Figure 9:**
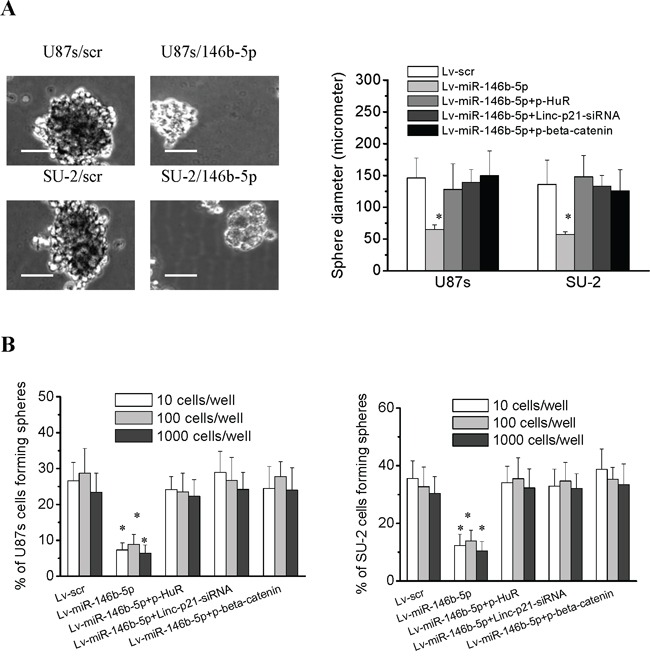
MiR-146b-5p overexpression decreased the neurosphere formation capacity of GSCs **A.** The diameters of neurospheres of GSCs with miR-146b-5p overexpression plated at 100 cells per well in 96-well plates. *P<0.01 vs Lv-scr. Representative images of neurospheres (light microscope). Scale bar =50 μm. **B.** The percentage of GSCs with miR-146b-5p overexpression forming neurospheres. *P<0.01 vs Lv-scr.

GSCs were seeded into 96-well plates at different density. After 12 d culture, diameters of GSCs/Lv-miR-146b-5p neurospheres were reduced compared with those of GSCs/Lv-scr (Figure 9A), and cells forming neurospheres were also reduced (Figure 9B). The results showed that miR-153 overexpression decreased GSCs neurosphere formation. After p-HuR, p-β-catenin and lincRNA-p21-siRNA transfection, neurosphere diameter and the percentage of cells forming neurospheres of GSCs/Lv-miR-146b-5p showed no difference compared with that of GSCs/Lv-scr, which suggested that knock-down lincRNA-p21 or HuR and β-catenin overexpression could rescue the neurosphere formation capacity of GSCs with miR-146b-5p overexpression.

### MiR-146b-5p overexpression decreased GSCs stemness and induced differentiation

**Figure 10:**
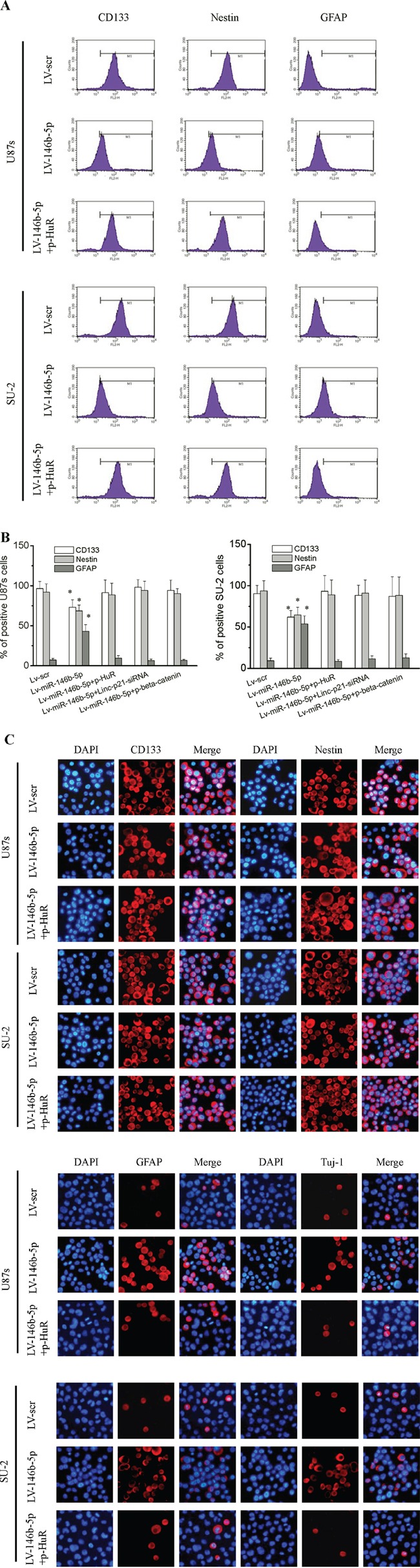
MiR-146b-5p overexpression decreased stemness and induced differentiation in GSCs **A.** Flow cytometry analysis of the expression of stem cell markers CD133 and nestin and differentiation marker GFAP. **B.** The percentage of positive GSCs. *P<0.01 vs Lv-scr. **C.** Immunofluoresence staining for CD133, nestin, GFAP and Tuj-1.

Flow cytometry analysis showed that stem cell markers CD133 and nestin expressions in GSCs/Lv-miR-146b-5p significantly decreased (*P*<0.01) compared with those of GSCs/Lv-scr, while the differentiation marker glial fibrillary acidic protein (GFAP) increased (Figure 10A and 10B; *P*<0.01). GSCs were also stained with immunofluoresent method for CD133, nestin, GFAP and Tuj-1. Representative photomicrographs showed that miR-146b-5p decreased CD133 and nestin expression and increases GFAP and Tuj-1expression (Figure 10C). Our results indicated that miR-146b-5p overexpression decreased GSCs stemness and induced differentiation. After p-HuR, p-β-catenin and lincRNA-p21-siRNA transfection, stem and differentiation markers of GSCs/Lv-miR-146b-5p showed no significant difference compared with that of GSCs/Lv-scr, which suggested that knock-down lincRNA-p21 or HuR and β-catenin overexpression could rescue the induced differentiation of GSCs owing to miR-146b-5p overexpression.

### MiR-146b-5p overexpression increased radiosensitivity of GSCs

**Figure 11:**
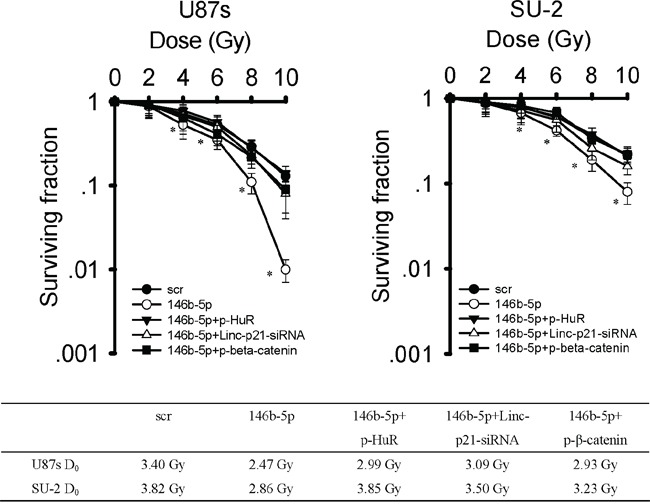
MiR-146b-5p overexpression decreased radioresistance of GSCs After exposure to 0, 2, 4, 6, 8 and 10 Gy X-ray irradiation, cell survival fractions were examined and the survival curves of cells were obtained from data fitting according to the linear quadratic model. Error bars indicate the standard error of the mean of three individual experiments. *P<0.01 vs U87s/Lv-scr or SU-2/Lv-scr. D_0_ (mean lethal dose) was calculated by the linear quadratic model.

After exposed to various dose of X-ray, GSCs survival curves, as shown in Figure 11, were obtained. It is clear that GSCs with miR-146b-5p overexpression were more radiosensitive than GSCs with scramble sequence integration. After p-HuR, p-β-catenin and lincRNA-p21-siRNA transfection, radiosensitivity of GSCs/Lv-miR-146b-5p decreased. These results indicated that knock-down lincRNA-p21 or HuR and β-catenin overexpression could rescue the decreased radioresistance resulting from miR-146b-5p overexpression in GSCs.

### MiR-146b-5p overexpression reduces tumorigenic capacity of GSCs *in vivo*

**Figure 12:**
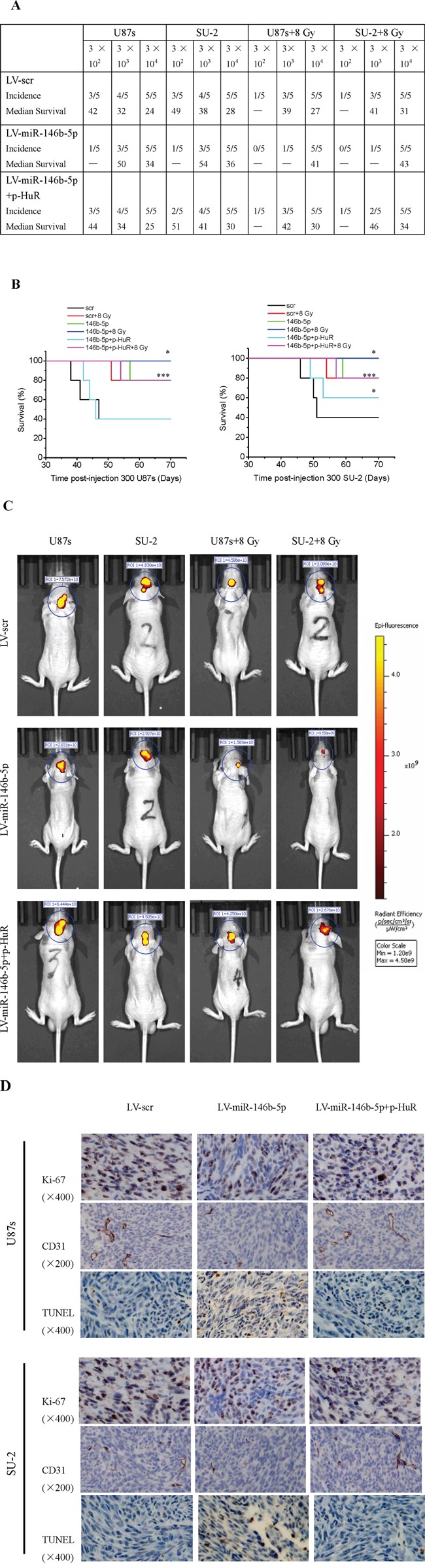
MiR-146b-5p overexpression reduces tumorigenic capacity of GSCs **A.** Tumor incidence of GSCs and median survival of nude mice bearing human GSCs. **B.** Kaplan-Meier curves of mice bearing human GSCs. *P<0.05 vs Lv-scr. **C.** Photon measurement around the tumor area showed by *in vivo* imaging system (IVIS). **D.** Ki-67, CD31 and TUNEL staining of tumor sections.

Orthotopic transplantation is a golden standard to determine tumor-initiating capacity of cancer stem cells. To explore the effect of miR-146b-5p overexpression on tumor formation, GSCs stably expressing miR-146b-5p mature sequence or scramble sequence were intracranially implanted into immuno-compromised hosts. Tumor-bearing mice were monitored daily until neurologic signs occurred in each animal. When 300 or 3,000 cells were injected, the tumor incidence of GSCs with miR-146b-5p overexpression decreased compared with that of GSCs expressing scramble sequence, and the tumor incidence of GSCs decreased after 8 Gy X-ray irradiation (Figure 12A). After transfection with p-HuR, the tumor incidence of GSCs with miR-146b-5p overexpression increased. Survival of mice with GSCs overexpressing miR-146b-5p increased compared to that with GSCs expressing scramble sequence. Nevertheless, survival of mice with GSCs overexpressing miR-146b-5p and HuR decreased compared with that of GSCs overexpressing miR-146b-5p. Kaplan-Meier curves also demonstrated increment in survival with the coming of miR-146b-5p overexpression and/or 8 Gy X-ray irradiation (Figure 12B). Three weeks after 3×10^4^ GSCs transplanted, the photon measurement around the tumor area showed by *in vivo* imaging system (IVIS) decreased with introduction of miR-146b-5p overexpression and/or 8 Gy X-ray irradiation (Figure 12C). In microscopic observation of tumors, lesser number of Ki-67 and CD31-positive cells and more number of TUNEL-positive cells were observed in miR-146b-5p overexpression group compared with scramble sequence group, while the number of Ki-67, CD31 and TUNEL-positive cells in miR-146b-5p and HuR overexpression group showed no significant changes compared with scramble sequence group (Figure 12D). The results indicated that miR-146b-5p overexpression may result in glioma growth delay through reducing cell proliferation and angiogenesis and inducing apoptosis. These *in vivo* data suggested that miR-146b-5p overexpression could reduce tumorigenic capacity of GSCs and increase survival in mouse models of human glioma.

## DISCUSSION

MiR-146b-5p expression has been found in almost all human organs [38]. In papillary thyroid carcinoma and lung cancer, it acts as an oncogene and has been regarded as a relevant diagnostic marker [39, 40]. However, in glioma and some other cancers, miR-146b-5p expression is downregulated and acts as an tumor suppressor gene [41]. A recent study revealed that miR-146b-5p inhibited proliferation and promoted apoptosis of glioma, and predicted better prognosis in human gliomas, especially in glioblastoma. Its downregulation was a main reason of glioma genesis and malignant progression [42]. Consistent with these results, we found miR-146b-5p was down-regulated in GSCs compared to non-GSCs glioma cells and its overexpression could suppress stemness and radioresistance of GSCs by targeting HuR, which was verified as a miR-146b-5p target in GSCs by bio-informatic analysis, real-time PCR, and luciferase reporter assay in the present study.

HuR, a ubiquitous RNA binding protein (RBP), influences cell proliferation, survival and carcinogenesis through its RNA recognition motifs binding to adenine and uridine (AU)-rich stability elements (ARE) in 3′-UTR of mRNA [43]. Instead of acting as an RNA stabilizer, HuR works with the Ago2 protein and let-7 to destabilize lincRNA-p21 [22]. Many studies have reported elevated expression of HuR in numerous malignancies [44]. In this study, we found that HuR expression was up-regulated in GSCs compared with non-GSCs glioma cells, which resulted in lincRNA-p21 down-regulation, leading to elevated β-catenin expression in GSCs. The mRNA of c-myc, a target gene of β-catenin, has been verified as a target of HuR [45]. Kim et al. further revealed that the site bound by HuR are proximal to that bound by let-7 in 3′-UTR of c-myc, which suggested that HuR might recruit let-7-loaded RNA-induced silencing complex to inhibit c-myc expression in HeLa cells [46]. Thus, the effect of HuR on c-myc expression might be direct binding c-myc mRNA and supressing translation in cervical carcinoma, besides its indirect influence through lincRNA-p21/β-catenin revealed in this study.

In order to target the aberrant HuR/lincRNA-p21/β-catenin pathway in GSCs, We employed lentiviral vector to overexpress miR-146b-5p and analyzed GSCs phenotypic changes. We found that miR-146b-5p overexpression increased apoptosis and radiosensitivity, and decreased cell viability, neurosphere formation capacity and stem cell marker expression in GSCs. Moreover, knock-down lincRNA-p21 or HuR and β-catenin overexpression could rescue the phenotypic changes resulting from miR-146b-5p overexpression in GSCs. These results demonstrated that miR-146b-5p played a vital role in GSC stemness, survival, and radioresistance *in vitro*, so we further evaluated the ability of miR-146b-5p overexpression to improve the survival of immuno-compromised mice bearing human GSCs. The *in vivo* data showed that miR-146b-5p overexpression could reduce tumor-initiating potential of GSCs and increase survival in mice bearing human GSCs.

In conclusion, β-catenin signaling pathway is frequently activated through the miR-146b-5p/HuR/lincRNA-p21 axis in GSCs, which contributes to stemness and radioresistance of GSCs. Our data suggest that targeting the miR-146b-5p/HuR/lincRNA-p21/β-catenin signaling pathway may be valuable therapeutic strategies against glioma.

## MATERIALS AND METHODS

### Patient samples

The present study consisted of 37 glioma tissues and 8 normal brain tissues, which were resected at the department of neurosurgery of the first affiliated hospital of Jilin University between 2013 and 2014. None of the patients received preoperative treatment such as irradiation or chemotherapy. All research protocols in the present study were approved by the Ethics Committee of the first affiliated hospital of Jilin University.

### Cell culture

Normal human astrocytes (NHA, ScienCell Research Laboratories, Carlsbad, CA) were cultured in a growth medium containing 3% fetal bovine serum, 25 μg/ml bovine insulin, 20 ng/ml epidermal growth factor (EGF), 20 ng/ml progesterone, and 50 μg/ml transferrin at 37°C in 5% CO_2_, 90% relative humidity. The human glioma cell lines U87 were purchased from the Type Culture Collection of the Chinese Academy of Sciences and cultured in DMEM/F12 medium (1:1, Hyclone) supplemented with 10% fetal bovine serum (FBS) (Invitrogen) in a humidified atmosphere containing 5% CO2 at 37°C. The human GSCs culture U87s, derived from glioma cell line U87, were enriched using serum-free clone formation method with stem cell medium, which has been previously described [33]. The patient-derived GSCs culture SU-2 and non-GSCs glioma cell culture NSSU-2, isolated from a surgical specimen of a 52-year-old female patient with glioblastoma, were kindly provided by Dr. Ting Sun in neurosurgery laboratory of the first affiliated hospital of Soochow University [33, 47, 48]. GSCs were cultured as spheres in serum-free DMEM/F12 supplemented with 2% B27 Neuro Mix (invitrogen), 20 ng/mL epidermal growth factor (EGF) and 10 ng/mL basic fibroblast growth factor (bFGF) (PeproTech). The cultures were fed every 3 days with one-third volume of fresh medium. Cell passaging was performed by dissociation of spheres using NeuroCult chemical dissociation kit (StemCell Technologies). Neurospheres of around 8-12 passages were used for this study.

### Real-time reverse transcription-polymerase chain reaction (RT-PCR)

Total RNA from brain tissues and glioma cells and tissues were extracted using TRIzol (invitrogen), and cDNA was generated using the First Strand cDNA Synthesis kit (Roche), according to the manufacturer's instructions. mRNA expression was analyzed using the SYBR Green PCR Master Mix (Applied Biosystems) on a 7300 Real-Time PCR System (Applied Biosystems, Foster City, CA) using standard conditions as previously described [33]. Fold changes in mRNA expression were quantified with the 2^−ΔΔCT^ relative quantification method using β-actin as house keeping control. The primer sequences used were as follows: LincRNA-p21 forward, 5′-CCATCCACCTACATCACGAAG-3′ and reverse, 5′-CTCTGGAGAACCCCGACTAAT-3′. β-catenin forward, 5′-ATTGTCCACGCTGGATTTTC-3′ and reverse, 5′-TCGAGGACGGTCGGACT-3′. C-Myc forward, 5′-CGCTTCTCTGAAAGGCTCTCCTTG-3′ and reverse, 5′-GAGTCGTAGTCGAGGTCATAGTTC-3′. Cyclin D1 forward, 5′-AGGAGAACAAACAG ATCA-3′ and reverse, 5′-TAGGACAGGAAGTTGTTG-3′. HuR forward, 5′-AGACATGTTCTCTCGGTTTG-3′ and reverse, 5′-ACTGAACCTGACCGTACAACTGGTAATTGCCTCTTCTG-3′. MiRNA detection by real-time analysis involved reverse transcription of cDNA using a small RNA specific stem-loop RT primer. Once specific cDNA was generated, individual miRNA expression was assessed by real-time PCR according to the TaqMan MicroRNA Assay. Results were normalized to small nuclear RNA U6 that served as control and the data were expressed as Log 2 fold change in respective miR/U6 snRNA levels.

### Western blot analysis of protein expression

Western blotting was performed using standard procedures. The following primary antibodies were used: mouse monoclonal anti-β-catenin, c-myc, Cyclin D1, Hu antigen R (HuR), Phospho-AKT, Total-AKT, mammalian target of rapamycin (mTOR), B-cell-specific Moloney murine leukemia virus insertion site 1 (Bmi1), glial fibrillary acidic protein (GFAP), Lamin b1 and β-actin (Santa Cruz Inc. California, USA). Experiments were repeated three times. The relative levels of protein expression were normalized against protein levels of an internal control gene, β-actin, performed in the same run.

### Subcellular fractionation

Cells were homogenized in hypotonic buffer (10 mM Tris-HCl, pH 7.8, 150 mM NaCl, and 1 mM EDTA) containing 0.1% (w/v) Triton X-100. The lysates were centrifuged at 3000 rpm for 10 min at 4°C. The pellet was re-suspended in hypotonic buffer and re-centrifuged and was used as the nuclear fraction. The supernatant fraction was re-centrifuged at 16,000 rpm for 20 min at 4°C and used as the cytoplasmic fraction.

### Chromatin immunoprecipitation

Cells were crosslinked with 1% formaldehyde, harvested, resuspended in lysis buffer supplemented with 1 mM PMSF, and sonicated. The chromatin fragments were immunoprecipitated using either p53 or IgG (Millipore, MA, USA) antibodies. After extensive washing steps, the formaldehyde crosslinks were reversed by treatment at 65°C. DNA was purified by phenol/chloroform extraction followed by ethanol precipitation and samples were used in real-time PCR.

### siRNA design and transfection

The cDNA sequences of AKT and mTOR were obtained from GenBank (NM_001014431 and 004958). The siRNA target design tools from Ambion were used to design siRNA. AKT-siRNA was designed and synthesized as follows: sense: 5′-CGGUAGCACUUGACCUUUUTT-3′, antisense: 5′-AAAAGGUCAAGUGCUACCGTG-3′. mTOR-siRNA was designed and synthesized as follows: sense: 5′-CAUUCGCAUUCAGUCCAUATT-3′, antisense: 5′-UAUGGACUGAAUGCGAAUGAT-3′. The sequence of LincRNA-p21 was previously reported [18] and LincRNA-p21-siRNA was designed and synthesized as follows: sense: 5′-UGAAAAGAGCCGUGAGCUATT-3′, antisense: 5′-UAGCTCACGGCUCUUUUCAAT-3′ (Sangon Inc. Shanghai, China). Cells were plated 24 h prior to transfection and were transfected in 6-well plates by use of Lipofectamine RNAiMAX (Invitrogen, Carlsbad, CA, USA). AKT, mTOR and LincRNA-p21-siRNA and negative control siRNA (nc-siRNA) were used at 100 nM final concentration.

### Luciferase reporter assay

The 3′UTR of HuR gene contains a putative miR-146b-5p-target site: 5′-AAGAUUAACCCUC AAAGUUCUCU-3′. The wild type and mutated 3′UTRs of HuR were amplified by PCR from cDNA of U87s cells and were ligated into the pGL3 basic luciferase expression vector (pGL3-Luc) (Promega, Madison, WI, USA) at the 3′-end of the luciferase coding sequence. The pGL3-Luc vector containing wild type and mutated 3′UTRs of HuR and the internal control vector pRL-TK (Promega) were co-transfected into U87s and SU-2 cells. Twenty four hours later, 20 nM of miR-146b-5p oligos (5′-UGAGAACUGAAUUCCAUAGGCU-3′) or scramble oligos (5′-UUCUCCGAACGUGUCACGUTT-3′) (GenePharma Co. Shanghai, China) were transfected into U87s and SU-2 cells. Luciferase activity was measured 48 h after vectors transfection using Dual-luciferase Reporter Assay System (Promega) according to the manufacturer's instructions.

### Generation of stable cell lines

GSCs with stable integration of the miR-146b-5p mature sequence or scramble sequence were generated through lentiviral-mediated gene transfer [33]. To generate the respective viruses, 293T cells were transfected with the lentiviral vector, pGLV-miR-146b-5p-GFP or pGLV-scr-GFP, and the packaging plasmid PG-P1-VSVG, PG-P2-REV and PG-P3-RRE according to standard protocols. The target GSCs were infected with viruses encoding either miR-146b-5p or scramble sequence and selected using puromycin. After 4 weeks, single clones were analyzed for positive green fluorescent protein (GFP) signals. The positive clones were expanded for additional testing.

### mRNA decay studies

Actinomycin D (Sigma, St Louis, MO, USA) was added to the media at a concentration of 5 μg/ml to arrest transcription at time zero. Cells were harvested for RNA analysis at 0, 1, 2, and 3 h. The half-life of each mRNA was calculated using the following formula: t_1/2_ = ln2/k_decay_. The decay rate constant (k_decay_) was obtained from fitting the changes of mRNA concentration with respect to time to a first order decay model using a least squared differences method [49].

### LincRNA-p21, HuR and β-catenin expression vectors construction and transfection

Full-length cDNA of LincRNA-p21, HuR and β-catenin were isolated from cDNA of U87s cells and were amplified through RT-PCR using specific primers. The primer sequences used were as follows: LincRNA-p21 forward, 5′-TGGCAGTCTGACCCACACTCCCCACGCCC-3′ and reverse, 5′-ACAGTGCACAGACAATCATACACACGTGT-3′. HuR forward, 5′-ATGGTCTAA TGGTTATGAAGAC-3′ and reverse, 5′-TTTGTGGACTTGTTGGTTTTG-3′. β-catenin forward, 5′-AAAATCCAGCGTGGACAATGG-3′ and reverse, 5′-TGTGGCAAGTTCTGCATCATC-3′. The amplified cDNA fragment were sub-cloned into the pcDNA3.1(+) vectors. The recombinant vectors were confirmed by the digestion analysis of restriction endonuclease and inserted sequences were verified by DNA sequencing. Cells were transfected in 6-well plates by use of Lipofectamine 2000 (Invitrogen, Carlsbad, CA, USA).

### Cell viability assay

GSCs viability was measured by a 3-(4, 5-dimethylthiazol-2-yl)-2, 5-diphenyl tetrazolium bromide (MTT) assay (Sigma, St Louis, MO, USA) after 2, 4 and 6 days culture. GSCs viability was calculated as a ratio of OD value, and the cell viability of GSCs/Lv-scr after different periods of culture were taken as 100%.

### Flow cytometric analysis of cell cycle and apoptosis

GSCs were harvested and fixed overnight with 70% ethanol at 4°C, followed by resuspension in 500 μL of PBS. After addition of 10 μL RNase (10 mg/mL), cells were left for 30 minutes at 37°C and stained with 10 μL propidium iodide (1 mg/mL). Cellular DNA content was determined on a flow cytometer Beckton Dickinson (*BD*) *FACScan* (*BD* Biosciences, San Jose, CA). Quantification of apoptotic cells was performed according to the Annexin-V-PE/7-AAD Apoptosis Detection Kit manufacturer instructions (KeyGen Biotech. Nanjing, China). Analyses were performed by a flow cytometer (BD FACScan). Phycoerythrin (PE) -positive and 7-amino-actinomycin D (7-AAD) -negative cells were regarded as apoptotic cells.

### Neurosphere formation assay

GSCs were plated at 10, 100 or 1000 cells per well in 96-well plates. After culture for 12 d, the number of neurospheres that contained more than 20 cells in each well was determined, and neurosphere formation rate was calculated as the number of neurospheres/100 × 100%.

### Flow cytometry analysis and immunofluoresence staining for the stem cell markers and the differentiation markers

For flow cytometry analysis, GSCs enriched in DMEM/F12 medium supplemented with growth factors were dissociated, washed, and incubated with PE-conjugated CD133, nestin and GFAP antibody (Milteny Biotech) in phosphate-buffered saline (PBS)–BSA for 1 h. Labeled cells were resuspended in PBS with 1% FBS, and analyzed by a flow cytometer Beckton Dickinson (*BD*) *FACScan* (*BD* Biosciences, San Jose, CA). Isotypic IgG and unstained cells served as negative controls.

For immunofluoresence staining analysis, GSCs were fixed in 4% paraformaldehyde (Sigma-Aldrich) for 15 minutes at room temperature, permeabilized using 0.1% Triton-X100 (Sigma-Aldrich) for 20 min and blocked in 5% Bovine serum albumin (BSA) (Sigma-Aldrich) for 1 h at room temperature. Then the GSCs were immunostained with mouse antibodies against human CD133 (1:200, Santa Cruz), nestin (1:300, Santa Cruz), glial fibrillary acidic protein (GFAP, 1:500, Abcam) and Tuj-1 (1:500, Abcam) for 45 min in darkness. Subsequent visualization for the stem cell markers, CD133 and nestin, and the differentiation markers, GFAP (astrocytes) and Tuj-1 (neurons), were performed with Texas Red-conjugated anti-mouse IgG (1:1000, Vector Laboratories) for 30 min at room temperature and the nuclei were counterstained with 4′,6′-diamidino-2-phenylindole (DAPI, 1:500, Santa Cruz). Fluorescence images were captured with fluorescence microscope (Olympus BX50).

### Clonogenic cell survival assay

Cells were irradiated with X-ray (6 MV, the dose rate was 200 cGy/min) by a PRIMUS accelerator (SIEMENS Medical Solutions, Erlangen, Germany) at room temperature. After irradiation, a specific number of cells (100 for cells irradiated with 0 or 2 Gy, 200 for 4 Gy and 2000 for 6, 8 and 10 Gy) was plated in petri dishes in triplicate for clonogenic assay. Then the cells were incubated for 12 days. Colonies were fixed by 37% formaldehyde solution and stained with crystal violet and colonies of more than 50 cells were counted. Furthermore, the cell survival fraction was counted out and the mean lethal dose (D_0_) was calculated by the linear quadratic model.

### Orthotopic transplantation assays

For intracranial implantation, 36 h after irradiation with 8 Gy X-ray, GSCs/Lv-scr, GSCs/Lv-miR-146b-5p and GSCs/Lv-miR-146b-5p+p-HuR cells were counted and the indicated numbers of GSCs were implanted into the right frontal lobes of 6–8-week-old female athymic nude mice (Experimental Animals Center of Shanghai Institute of Life Science, Shanghai, China).

Three weeks later, the Xenogen *in vivo* imaging system (IVIS) 50 system was used to visualize tumors, and photon measurement was defined around the tumor area and quantified using Living Image software (Caliper Life Science SA, Villepinte, France). The mice used for immunohistochemical studies were sacrificed three weeks after implantation. Tumor tissues were fixed and imbedded in paraffin. Tumor sections of 5 μm were cut from the embedded tissue and incubated with specific primary antibodies, including rabbit polyclonal antibody to human Ki-67 (KeyGen Biotech.) and mouse CD31 (eBioscience, Inc., San Diego, CA, USA) for 1 h at 37°C followed by overnight at 4°C in humidity chamber. Negative controls were incubated only with universal negative control antibodies under identical conditions. The sections were then incubated with appropriate biotinylated secondary antibody for 60 min at room temperature. Thereafter, sections were incubated with conjugated horseradish peroxidase streptavidin (KeyGen Biotech.) for 60 min, followed with 3,3′-diaminobenzidine (Sigma) working solution, and counterstained with hematoxylin. Apoptotic cells in tumor tissues were detected by terminal deoxynucleotidyl transferase (TdT)-mediated dUTP-biotin nick end labeling (TUNEL) stain, using an In Situ Cell Death Detection Kit (KeyGen Biotech.) following the manufacturer's specifications. All the animal experiments were conducted in accordance with Guidelines for the Welfare of Animals in Experimental Neoplasia.

### Statistical analysis

All statistical parameters were calculated with GraphPad Prism 6.01 (GraphPad Software Inc.). Student's t test was used for most data analysis. For comparisons among more than two groups, One-way Analysis of Variance (ANOVA) followed by Bonferroni post-test was performed. Mice survival were evaluated by the Kaplan-Meier method and analyzed by the log-rank test. *P*<0.05 was considered to be statistically different.
